# Impact of built environment change on all-cause and cause-specific mortality: a novel longitudinal method and study

**DOI:** 10.1136/jech-2023-220681

**Published:** 2023-06-27

**Authors:** Laura Macdonald, Natalie Nicholls, Denise Brown, Richard Mitchell

**Affiliations:** MRC/CSO Social and Public Health Sciences Unit, University of Glasgow, Glasgow, UK

**Keywords:** GIS, MORTALITY, Health inequalities, PUBLIC HEALTH

## Abstract

**Background:**

Public health research increasingly acknowledges the influence of built environments (BE) on health; however, it is uncertain how BE change is associated with better population health and whether BE change can help narrow health inequalities. This knowledge gap is partly due to a lack of suitable longitudinal BE data in most countries. We devised a method to quantify BE change longitudinally and explored associations with mortality. The method is replicable in any nation that captures BE vector map data.

**Methods:**

Ordnance Survey data were used to categorise small areas as having no change, loss or gain, in buildings, roads, and woodland between 2015 and 2019. We examined individual mortality records for 2012–2015 and 2016–2019, using negative binomial regression to explore associations between BE change and all-cause and cause-specific mortality, adjusting for income deprivation.

**Results:**

BE change varied significantly by deprivation and urbanicity. Change in the BE and change in mortality were not related, however, areas that went on to experience BE change had different baseline mortality rates compared with those that did not. For example, areas that gained infrastructure already had lower mortality rates.

**Conclusion:**

We provide new methodology to quantify BE change over time across a nation. Findings provide insight into the health of areas that do/do not experience change, prompting critical perspectives on cross-sectional studies of associations between BE and health. Methods and findings applied internationally could explore the context of BE change and its potential to improve health in areas most in need beyond the UK.

**WHAT IS ALREADY KNOWN ON THIS TOPIC**

- Cross-sectional public health research indicates associations between non-communicable diseases and the built environment (BE), while longitudinal data about people’s lives and their health has proved useful in understanding how health inequalities have developed over time.

**WHAT THIS STUDY ADDS**

- To understand the role of the BE in protecting or harming health, we also need longitudinal data on the BE linked to data on individuals.
- We quantified BE change over time and explored associations with change in mortality rates across Scotland; this is the first study to do so on a national scale.
- We found that it was the type of place that was targeted for (re)development, rather than the (re)development itself, that was related to population health; our methods could be applied anywhere that has repeated vector mapping data of the BE.

**HOW THIS STUDY MIGHT AFFECT RESEARCH, PRACTICE OR POLICY**

- If we understand more about the context of BE change, and which features of BE change are beneficial to health, then we can use policy and planning to manipulate and direct change to the populations and areas most in need. Understanding that health may already be better in areas that gain (re)development prompts critical perspectives on studies of the BE and health associations.

## Introduction

The built environment (BE) encompasses human-made physical features in which people live, work, interact, etc^1^ including buildings, roads and created and managed green spaces such as urban parks.^2^ Public health research increasingly acknowledges the influence the BE has on health and well-being in all settings; its characteristics can be of benefit or harm.^1 3–5^ We developed and tested a method to provide evidence for the consequences of BE change on population health.

Much of the existing international research focuses on specific BE attributes, such as transport infrastructure, and its associations with health. Few studies consider multiple aspects of the BE simultaneously. This is surprising since major BE interventions almost always alter multiple aspects of the BE. You cannot, for example, build a major new housing development without adding roads, and buildings and gardens, etc, and whatever environment previously existed on the site will be largely lost in the development. In this study, therefore, we made progress in considering multiple aspects of the environment together, that is, building/road infrastructure and woodland. Here, we rely on these specific BE measures as indicators of neighbourhood change overall as, although the BE consists of multiple features, not all are captured by data which are comprehensive and comparable over time. These elements of the BE are common concerns in local planning processes (eg, with loss of woodland always a contested issue), but the choices were also grounded in evidence that each aspect can affect health. In the UK-based research, for example, higher densities of road junctions in cities were associated with higher all-cause mortality and circulatory mortality; poorer air quality being a likely contributory factor.^6^ Similarly, a study in Canada found positive associations between road length and all-cause mortality risk, and between road density and mortality risk (eg, from circulatory and respiratory diseases).^7^ Research on the health impacts of building density, in particular housing density, is inconclusive. Impacts may be context dependent. For example, increased housing density with proximal amenities might increase active travel and contribute to reduced air pollution, but health benefits may be hindered if housing density is increased close to high traffic roads.^8^ High density housing has been associated with lower all-cause mortality in Australia,^9^ but, contrarily, linked to higher mortality rates in Western European.^10^ Considerable research identifies associations between green space and lower all-cause^11–14^ and cause-specific mortality,^11 15^ while growing evidence supports the positive health impacts of treed environments specifically, in relation to reduced risk of non-communicable diseases.^16^

Existing studies rely on cross-sectional measures of the BE, partly due to the challenge of accessing comprehensive and accurate longitudinal BE data. The UK, like many nations, has excellent sources of longitudinal data about people’s lives and health, which are useful in understanding health, and health inequalities. These comprise major panel and internationally recognised cohort studies (eg, Growing up in Scotland, Understanding Society), in addition to, various Trusted Research Environment services providing secure access to administrative health data (eg, the Scottish Data Safe Haven, Office for National Statistics (ONS) Secure Research Service). To understand the BE’s role in protecting or harming health, however, we also need longitudinal BE data. By linking BE change measures to health data, we might understand which types of change affect area-level health and make recommendations to reduce inequalities. Links between BE and health inequalities have been postulated because the BE contains and shapes some of the wider determinants of health. Unequal access or exposure to these determinants is one pathway to health inequality.^17^ It also follows that planned BE change to improve and equalise these determinants of health might play a role in narrowing health inequalities, but without measuring BE change and observing impact we cannot be sure.

Existing longitudinal research on the health effects of BE change tends to be small-scale, studying targeted urban renewal within poorer neighbourhoods. Renewal was associated with no/slight health improvements in the UK-based studies,^18^ in European countries such as the Netherlands^19^ and Spain,^20^ and further afield.^21^ One study measured change in the BE within selected neighbourhoods in Glasgow, Scotland, for example.^22^ Although the findings indicated that well-being worsened in areas with higher BE change, the study measured change more generally, without subcategorising specific change. In our study, we extend and develop fine spatial scale measures of change to cover a whole country (Scotland), identify different types of change and seek association with population health. We created a methodology that would be replicable in other nations. Scotland is a suitable case study country as it has low life expectancy and high inequality relative to comparator nations. Additionally, it continues to experience BE change.

Here, we develop prepiloted techniques^23^ to create measures of BE change, in building/road infrastructure and in woodland, between 2015 and 2019 and, for the first time, combine this with routinely collected data on mortality to explore associations between change and mortality. We also explore change ‘context’ and whether areas targeted for change already had differing mortality levels at baseline. We focus on mortality from all-causes, chronic lower respiratory disease and circulatory disease. Respiratory and circulatory disease both have high burden in Scotland,^24^ and research suggests plausible aetiological links to the BE.^6 7 11 15^

Our research questions were: Was there a change in the BE over time, and, if so, where did change occur (eg, in more or less deprived, urban or rural, areas)? Did mortality (for all-causes, from chronic lower respiratory disease, and from circulatory disease) change over time, and were the mortality rates of the places that experienced BE change already different at baseline? Is change over time in the BE associated with change in mortality, and does the combination of specific kinds of change in infrastructure/woodland have an influence on any change that occurs?

## Methods

We created or obtained data on (1) local area and population characteristics, (2) BE change and (3) mortality. Our common spatial framework was data zones, the smallest level for which mid-year population estimates (used to calculate mortality rates) are available. Data zones are small spatial units used in statistics reporting and typically contain 500–1000 residents. They are designed to be socially homogeneous and delineate different neighbourhoods. The 2011 data zone boundaries (n=6976) were downloaded from the UK government open data website.^25^

### Local area characteristics

We used data zone-level Scottish Index of Multiple Deprivation (2016 SIMD) income quintiles,^26^ mid-2014 and mid-2018 Small Area Population Estimates from the National Records of Scotland (NRS),^27^ and urban/rural category as binary (2016).^28^

### BE measures

Ordnance Survey (OS) Open Map Local (OML) data were downloaded from EDINA Digimap for March 2015 (the earliest available data) and April 2019. OML is detailed, street-level, vector mapping data (as points, lines and polygons). ArcMap V.10.3 was used to create a grid of 500 metres squared (m^2^) cells covering Scotland,^23^ and building and woodland polygons, and road lines were overlaid with the grid. Where a building centroid/woodland centroid/road line section intersected a cell, it was linked to the cell’s unique ID. We calculated the area of buildings (m²), area of woodland (m²) and total length of road sections (metres) within each cell for both 2015 and 2019. For each year, we summed building and road values to create an ‘infrastructure’ measure; calculated infrastructure percentage coverage, and woodland percentage coverage, and calculated change between years for both.

**Figure 1:**
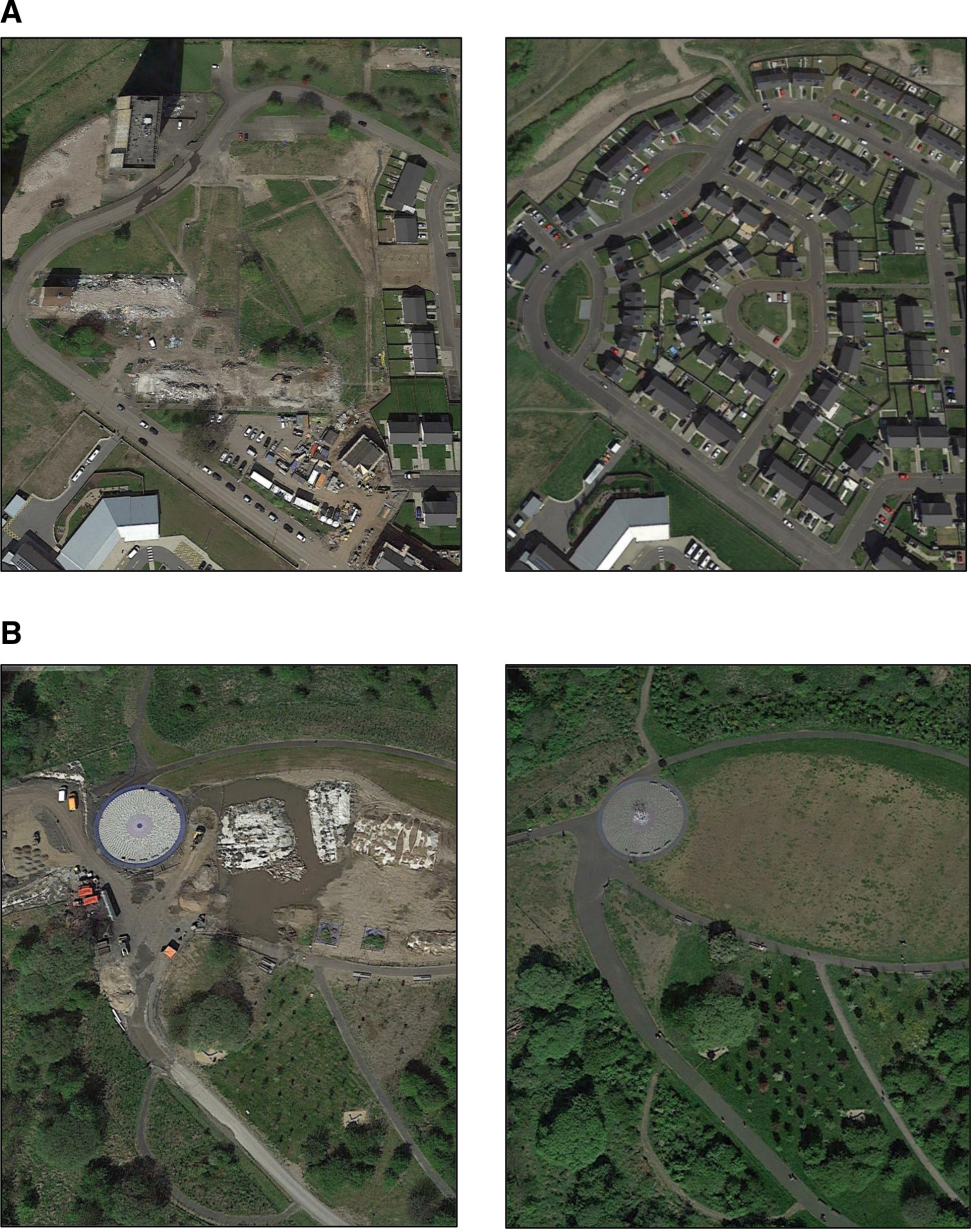
(A) Infrastructure increase, 2015–2019, in a Glasgow neighbourhood (Google Earth, 2023). (B) Woodland increase, 2015–2019, in a South Lanarkshire park (Google Earth, 2023).

For comparison with numeric change variables, categorical variables were also created (numeric variables not included in final models due to lack of associations). Woodland change was categorised into ‘1% or more loss’, ‘no change’, ‘1%–4.99% gain’, ‘5% or more gain’. Few data zones experienced infrastructure loss (<2%), therefore, infrastructure change was categorised into: ‘loss/little (no) change’ and ‘1% or more gain’. For woodland and infrastructure, little (or no) change included a gain or loss of up to 1%. The 1% threshold was used to exclude minor changes in features, which visual inspection of Google Earth imagery suggested were artefacts of data production rather than true ‘on-the-ground’ change. The 5% threshold was used as it has been previously considered a higher ‘dose’ of change.^22^ We tested the robustness of our findings in sensitivity analyses which compared different category cut-offs, for example, allowing any change, however, minimal, and varying the definitions of gain and loss categories. We ground truthed a sample of sites using longitudinal aerial imagery, to check methods realistically captured change (figure 1A,B).

### Mortality data

We obtained mortality records from NRS vital events data for every year from 2012 to 2019 by sex and age, for each data zone. Deaths were from ‘all causes’, ‘circulatory disease’ (eg, heart disease, stroke) as defined by ICD-10 codes I00–I99, and ‘chronic lower respiratory disease’ (eg, asthma, emphysema) as defined by ICD-10 codes J40–J47. Individual death records were aggregated into counts by sex-specific 5-year age bands, apart from neonates (age 0) and those aged over 85 (deaths at age 85 and over combined). To allow for random annual variations, these counts data were aggregated into two time periods: 2012–2015 and 2016–2019, corresponding approximately to timing of measurements of BE change.

### Statistical analyses

To investigate associations between BE change and mortality, two-level multilevel and single level negative binomial regression models were used, with the aggregated mortality count set as the outcome variable. For the multilevel models, data zone was set as the random effect (second level). Infrastructure and woodland change variables were included as fixed effects along with time period.

The log of the mid-year population estimates for 2013 and 2017, multiplied to adjusted for 4 years of aggregation were set as the offset. To evaluate any association with change in mortality, interactions between BE change and time period were included, as well as a three-way interaction between BE change type (ie, accounting for both infrastructure and woodland) and time. From this maximal model, the interaction terms were removed to simplify the model in order of terms (ie, three ways then two ways by largest p value). Single level models were used to evaluate whether there were differences in baseline mortality rates between areas that would undergo infrastructure and woodland change (ie, to explore the ‘context’ of BE change). All models were run for males and females separately, given their differences in mortality distribution, and all models controlled for age group, urban or rural location (BE coverage varies by urban/rural), and data zone-level SIMD income quintile. All analyses was performed in STATA 17^29^ (significance at 5%). Results are presented as mortality rate ratios (RR) and predicted mortality rates per 1000 population.

## Results

### Was there a change in the BE over time, and where did change occur (eg, in more or less deprived, urban or rural, areas)?

**Figure 2:**
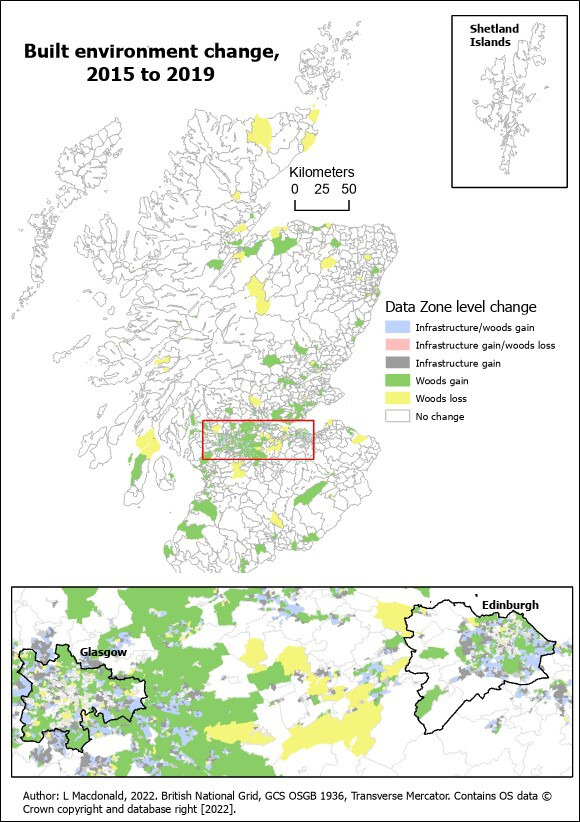
Built environment change, 2015–2019, within data zones across Scotland (GCS OSGB 1936, Geographic Coordinate System Ordnance Survey Great Britain 1936)

BE change occurred in some areas (figure 2, ie, the map (OS map data publishable via Open Government Licence)).^30^ Around 40.5% (n=2828) of data zones experienced no change in the BE, 12.0% (837) gained infrastructure and woodland, 22.2% (1551) gained infrastructure only, 15.1% (1055) gained woodland only, 5.1% (355) lost woodland only and 5.0% (350) gained infrastructure but lost woodland (table 1). Change varied by income quintile (χ^2^ p<0.001) and urban/rural (p<0.001). A greater percentage of poorer areas experienced no BE change (39%) than the least deprived (27.8%). A greater percentage of less deprived areas (18.7%) experienced gain in both woodland and infrastructure than in most deprived areas (8.4%). No change was more common in rural areas (68%) (urban 29%) (see online supplemental charts 1 and 2 for visualisations of the data).

10.1136/jech-2023-220681.supp1 Supplementary data

**Table 1:** Percentage of data zones showing change/no change in the BE over time, by SIMD income quintile (Q) and urban/rural

	% of data zones with loss/no change in infrastructure and			% of data zones with gain in infrastructure and		
	Loss of woodland	No change	Gain in woodland	Loss of woodland	No change	Gain in woodland
Income quintile						
Q1 (most deprived)	6.5	39.0	25.0	3.4	17.7	8.4
Q2	6.2	39.6	16.7	4.7	22.3	10.5
Q3	4.9	48.6	13.6	4.3	17.7	10.8
Q4	4.2	47.7	12.2	4.5	19.9	11.5
Q5 (least deprived)	3.6	27.8	8.1	8.2	33.6	18.7
Urban	4.6	28.8	17.3	6.0	27.4	15.9
Rural	6.3	68.4	10.0	2.7	9.9	2.7
Total	5.1	40.5	15.1	5.0	22.2	12.0

Quintiles 1–4 have 1395 data zones each; Q5 has 1396. 70.4% data zones are urban (n=4909).

BE, built environments; SIMD, Scottish Index of Multiple Deprivation.

### Did mortality (for all causes, from chronic lower respiratory disease and from circulatory disease) change over time?

There were 220 776 deaths from all causes across time period 2012–2015, and 230 487 deaths across time period 2016–2019. Multilevel models indicated that all-cause mortality rates did not differ between time points for males (p=0.070) nor females (p=0.57) (table 2). Circulatory disease mortality rates declined in males (RR 0.93, 95% CI 0.91 to 0.94, p<0.001) and females (RR 0.90, 95% CI 0.88 to 0.91, p<0.001). Chronic lower respiratory disease mortality rates declined in males (RR 0.94, 95% CI 0.90 to 0.97, p<0.001), but increased in females (RR 1.05, 95% CI 1.02 to 1.09, p=0.004) (table 2).

**Table 2:** Rate ratios (RR) of time-period association with mortality

	Male RR (95% CI)	P value	Female RR (95% CI)	P value
All causes	0.99 (0.98 to 1.00)	0.070	0.99 (0.99 to 1.01)	0.565
Circulatory	0.93 (0.91 to 0.94)	<0.001	0.90 (0.88 to 0.91)	<0.001
Respiratory	0.94 (0.90 to 0.97)	<0.001	1.05 (1.02 to 1.09)	0.004

### Were the mortality rates of the places that experienced BE change already different at baseline?

Some differences in mortality rates between areas that did, or did not, go on to experience BE change were evident at baseline (table 3). Areas that gained infrastructure already had lower all-cause mortality rates (males: RR 0.94, 95% CI 0.92 to 0.95, p<0.001, females: RR 0.94, 95% CI 0.93 to 0.96, p<0.001), as well as lower circulatory disease mortality rates (males RR 0.96, 95% CI 0.93 to 0.98, p=0.001; females RR 0.95, 95% CI 0.92 to 0.98, p<0.001) compared with areas with no change. Association between eventual infrastructure gain and lower rates of chronic lower respiratory disease was only found for males (RR 0.93, 95% CI 0.88 to 0.99, p=0.017). Associations with eventual woodland change were found at baseline for all-cause mortality rates (males p=0.018, females p<0.001). Post hoc tests showed that all-cause mortality rates were already higher in areas that would gain up to 5% woodland compared with areas with no change (males Šidák-adjusted p=0.017, females p=0.002). For females, areas with up to 5% woodland gain already had higher all-cause mortality rates than areas with loss (p<0.001). We explored average mortality rates per 1000 people across 2012–2019 and found differences in all-cause mortality rates by infrastructure/woodland change category. Regardless of woodland change, the highest mortality rates were found in areas with no infrastructure change and lowest in areas with gain (see online supplemental table 1).

10.1136/jech-2023-220681.supp2 Supplementary data

**Table 3:** Rate ratios (RR) of BE change comparing mortality rates at baseline (in 2012–2015)

			Male RR (95% CI)	P value	Female RR (95% CI)	P value
All causes	Infrastructure	Loss/no change (ref)		<0.001		<0.001
		1% or more gain	0.94 (0.92 to 0.95)		0.94 (0.93 to 0.96)	
	Woodland	No change (ref)		0.018		<0.001
		1% or more loss	0.99 (0.97 to 1.02)		0.98 (0.95 to 1.00)	
		1%–4.99%	1.03 (1.01 to 1.05)		1.04 (1.02 to 1.06)	
		5% or more gain	1.02 (0.99 to 1.05)		1.02 (0.99 to 1.05)	
Circulatory	Infrastructure	Loss/no change (ref)		0.001		<0.001
		1% or more gain	0.96 (0.93 to 0.98)		0.95 (0.92 to 0.98)	
	Woodland	No change (ref)		0.117		0.134
		1% or more loss	1.01 (0.96 to 1.05)		0.97 (0.93 to 1.01)	
		1%–4.99%	1.04 (1.01 to 1.07)		1.03 (0.99 to 1.06)	
		5% or more gain	0.99 (0.94 to 1.04)		1.00 (0.95 to 1.05)	
Respiratory	Infrastructure	Loss/no change (ref)		0.017		0.219
		1% or more gain	0.93 (0.88 to 0.99)		0.97 (0.92 to 1.02)	
	Woodland	No change (ref)		0.927		0.206
		1% or more loss	0.99 (0.91 to 1.09)		0.99 (0.91 to 1.81)	
		1%–4.99%	0.98 (0.91 to 1.05)		1.07 (1.00 to 1.13)	
		5% or more gain	0.99 (0.81 to 1.09)		1.02 (0.93 to 1.12)	

### Is change over time in the BE associated with change in mortality, and does the combination of specific kinds of change in infrastructure/woodland have an influence on any change that occurs?

There was no evidence of an association between BE change and mortality change, regardless of the combination of specific kinds of change examined, interactions with time were non-significant.

## Discussion

Our study findings indicated that BE change varied significantly by deprivation and urbanicity; that cause-specific (but not all-cause) mortality changed significantly over time; and the direction of change in the cause-specific mortality outcomes varied by sex. Change in the BE and change in mortality were not associated. Mortality rates of the places that went on to experience change were different at baseline; areas that would gain infrastructure had lower all-cause/cause-specific mortality rates. Baseline differences in mortality persisted over time, regardless of BE change.

We observed that more areas exhibiting change were urban and less deprived. This may reflect the locations where public and private investment is higher, where land is more available and where opportunities for commerce are greater. Scottish cities, as with many other developed nation urban contexts, continue to redevelop land in and around city centres, with policy supporting densification and brown field site (re)development. The location of these developments is intended to benefit sustainability, commerce and tourism.^31^ Our finding that areas that were going to gain infrastructure already had lower mortality rates at baseline perhaps signifies the targeting of more vibrant, already advantaged areas for (re)development. This is likely to be echoed in many other economically developed countries. A recent report, by the Scottish Land Commission, maintained that current approaches for new housing/land development were too focused on ‘shareholder value’, and ignored rural and more deprived areas.^32^ This is concerning as Scotland echoes other nations in its environmental injustice; those in the poorest areas are more likely to suffer exposure to an environment that negatively impacts their health^33^ and to die prematurely.^34^ Further research is needed to explore how/where BE change might be directed to benefit the health of those in deprived areas, and such research is vital as investment concentrated in advantaged areas has the potential to widen health inequalities. In methodological terms, such differences in mortality at baseline, between areas where environment changed and those where it did not, calls for critical reflection on findings within cross-sectional or uncontrolled longitudinal studies of BE and health.

A simple explanation for the lack of association between change in the BE and change in mortality may be that the period of time for change was not long enough, and thus the magnitude of change may be too small to translate to meaningful impact. We recognise that the aetiology of the diseases studied is long term. Future research could use similar methodologies to quantify change over 10, 20, or more years. Comparison with existing studies is difficult because so few explore interactions between change in several BE characteristics and associations with health, over a large geographical area. A UK study of deprived neighbourhoods’ change, via urban renewal, was linked to health improvements over 5 years.^35^ Renewal investment was assigned according to deprivation and population need. After receiving higher levels of investment, ‘higher need’ areas experienced, in relative terms, beneficial mental and physical health outcomes compared with ‘lower need’ areas. Within our study, we can only speculate on the potential positive health influence of BE change targeted specifically at areas with greater need; if there was a higher degree of change in the BE focused in deprived areas perhaps evidence of associations with change in mortality would emerge.

### Strengths and limitations

Our research exhibited several strengths. We devised a novel method to quantify BE change over time using OS data, the most detailed and accurate UK mapping product available. Our approach could be replicated in any nation that captures its BE in vector format. We used high-quality mortality and demographic data.^36^ We explored Scotland-wide change over time, existing research uses cross-sectional measures or is focused on small locales. We included infrastructure/woodland change interactions within our models, thus exploring the possibility that combinations of change offer differential impacts. Regarding limitations, the use of data zone-level analysis may lead to statistical bias from using arbitrarily classified units to report spatial patterning.^37^ Although we did ground truth a sample of areas, it is possible that changes detected in the data may not reflect change on the ground, particularly in terms of timing, and that our categories of change may simplify what change occurred in real terms. Furthermore, changes to both data zone definitions and the SIMD over time meant, we used the same deprivation measure for both time periods. We are limited by unavailability of OML road width measures; where widths changed (eg, minor roads to motorways) infrastructure change may be underestimated. We were unable to measure life-course BE which considered the migration of individuals in/out of areas, nor could we discern the length of time individuals resided in an area before death. These weaknesses lead to the possibility of selection and exposure misclassification biases. While disease aetiology is generally long term, BE change was measured over a relatively short time. This resulted in small change overall, and even where areas experienced greater change (5% or more) on-the-ground change may appear minimal. However, we believe that here we set foundations to explore longer-term associations (plan to do so via the ‘Built Environment Context and Change Atlas’).^38^ Finally, we recognise that our findings are Scotland specific and may not be generalisable elsewhere, however, our methods and findings could be applied internationally. Where longitudinal health and BE data is available, equivalent research beyond the UK, could explore the context of BE change and, where targeted, its’ potential to improve health in areas most in need. We acknowledge that replicating this methodology in countries without an authoritative geospatial organisation and longitudinal health data would be challenging.

## Conclusion

We devised a method to quantify BE change over time, contributing to methods to create longitudinal environmental data. This method is generalisable to any nation that captures its BE in vector data maps. Our findings provide insight into the context of BE change; with variation in the health of areas that did or did not experience change but also prompt critical perspectives on cross-sectional or uncontrolled studies of associations between BE and health.

Our findings have implications for policy and planning related to BE change for health improvement; the context of a BE intervention (ie, what kind of place it is) needs greater consideration, in addition to the type of change (ie, what happens to it). Greater emphasis should be attributed to the types of places (and populations) most in need of intervention, and investment in (re)development prioritised there.

## Data Availability

Data may be obtained from a third party and are not publicly available. Data were obtained from a third party and are not publicly available. Both datasets can be obtained via licence for this study and can be accessed from their primary sources; Ordnance Survey (https://beta.ordnancesurvey.co.uk/products/os-open-map-local) and National Records of Scotland (https://www.nrscotland.gov.uk/).
