# Supplementary material for: Impact of built environment change on all-cause and cause-specific mortality: a novel longitudinal method and study
Source: J Epidemiol Community Health. 2023 Jun 27;77(9):594–600. doi: 10.1136/jech-2023-220681 (PMC10423518; doi:10.1136/jech-2023-220681)

Supplemental charts 1 and 2.

Chart 1. Percentage of data zones with BE/no change by income deprivation quintile.

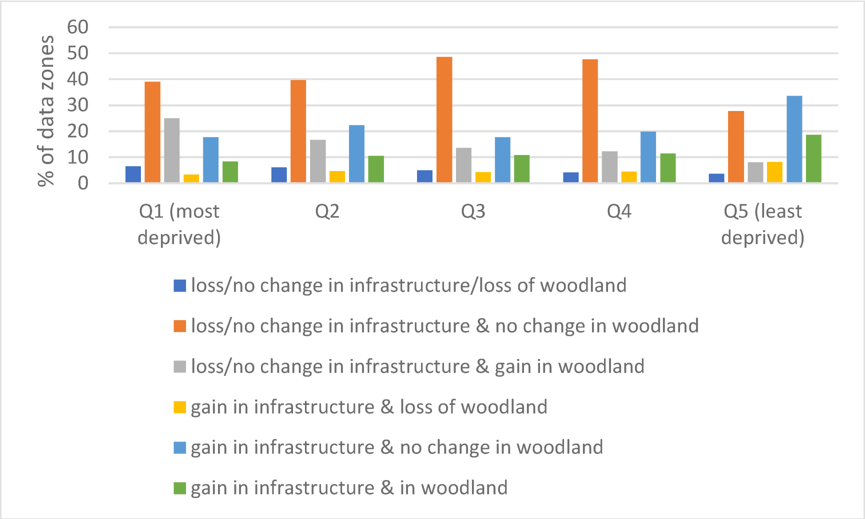

Chart 2. Percentage of data zones with BE/no change by urban/rural.

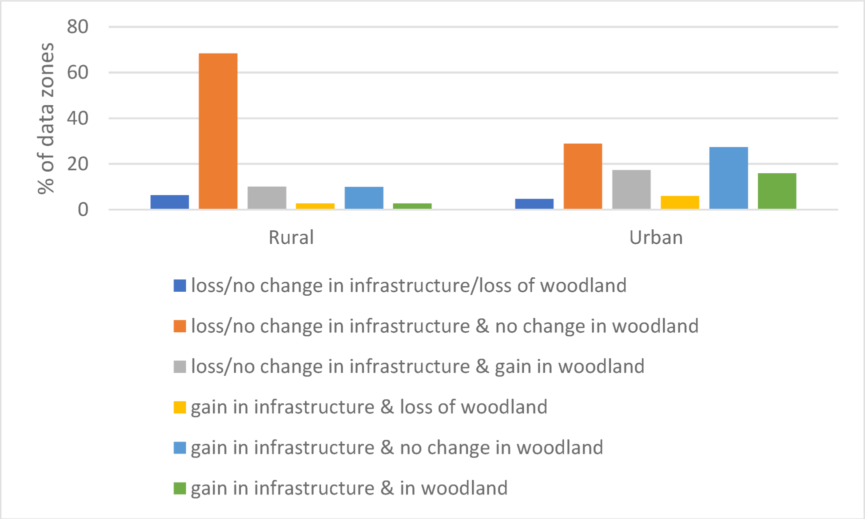

Supplement: Supplementary data [file jech-2023-220681supp001.pdf]
