# Supplementary material for: Impact of built environment change on all-cause and cause-specific mortality: a novel longitudinal method and study
Source: J Epidemiol Community Health. 2023 Jun 27;77(9):594–600. doi: 10.1136/jech-2023-220681 (PMC10423518; doi:10.1136/jech-2023-220681)
Supplement: Supplementary data [file jech-2023-220681supp002.pdf]

Supplemental table 1. Average mortality rates (2012-19) and 95% CIs for all-causes by BE change interactions

	Male : all-cause (95% CI)			Female: all-cause (95% CI)	
	Loss/no change in infrastructure	1% or more gain in infrastructure		Loss/no change in infrastructure	1% or more gain in infrastructure
**Woodland**					
**1% or more loss**	14.5 (14.2-14.8)	12.8 (12.5-13.1)		10.9 (10.7-11.1)	9.7 (9.5-9.8)
**No change**	13.9 (13.8-14.0)	13.3 (13.2-13.5)		10.8 (10.7-10.8)	10.3 (10.2-10.4)
**1-4.99%**	15.8 (15.6-16.0)	13.1 (12.9-13.3)		12.2 (12.0-12.3)	10.1 (10.0-10.3)
**5% or more gain**	16.3 (15.9-16.6)	13.7 (13.4-14.1)		12.6 (12.4-12.9)	9.9 (9.7-10.2)
